# Vaccines for COVID‐19

**DOI:** 10.1111/cei.13517

**Published:** 2020-10-18

**Authors:** J. S. Tregoning, E. S. Brown, H. M. Cheeseman, K. E. Flight, S. L. Higham, N.‐M. Lemm, B. F. Pierce, D. C. Stirling, Z. Wang, K. M. Pollock

**Affiliations:** ^1^ Department of Infectious Disease St Mary’s Campus Imperial College London London UK

## Abstract

Since the emergence of COVID‐19, caused by the SARS‐CoV‐2 virus at the end of 2019, there has been an explosion of vaccine development. By 24 September 2020, a staggering number of vaccines (more than 200) had started preclinical development, of which 43 had entered clinical trials, including some approaches that have not previously been licensed for human vaccines. Vaccines have been widely considered as part of the exit strategy to enable the return to previous patterns of working, schooling and socializing. Importantly, to effectively control the COVID‐19 pandemic, production needs to be scaled‐up from a small number of preclinical doses to enough filled vials to immunize the world’s population, which requires close engagement with manufacturers and regulators. It will require a global effort to control the virus, necessitating equitable access for all countries to effective vaccines. This review explores the immune responses required to protect against SARS‐CoV‐2 and the potential for vaccine‐induced immunopathology. We describe the profile of the different platforms and the advantages and disadvantages of each approach. The review also addresses the critical steps between promising preclinical leads and manufacturing at scale. The issues faced during this pandemic and the platforms being developed to address it will be invaluable for future outbreak control. Nine months after the outbreak began we are at a point where preclinical and early clinical data are being generated for the vaccines; an overview of this important area will help our understanding of the next phases.

## Graphical abstract

The spread of the virus SARS‐CoV‐2, the cause of COVID‐19 has triggered a tidal wave of vaccine development. In the first 9 months since the virus emerged over 200 vaccines have begun pre‐clinical development, 36 of which have entered clinical trials. This review will cover the platforms under assessment, the immune responses underpinning the vaccines, the results so far and the considerations for the next steps.

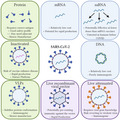

## Introduction

In November 2019, a cluster of pneumonia cases was detected in Wuhan, China [1]. These were the first cases of COVID‐19 caused by the novel beta‐coronavirus SARS‐CoV‐2. The genetic information was made publicly available on 10 January 2020, 54 days after the first declared case. Sixty‐three days after the SARS‐CoV‐2 sequence was published, on 13 March 2020, the first doses of the first human vaccine were being tested. By 24 September 2020, the SARS‐CoV‐2 vaccine landscape included 43 candidates being tested in clinical trials and more than 200 candidates. As the results from the Phase I trials and earliest Phase II/III trials emerge, this review will cover the platforms under development, the type of immune response required and the path to a clinical product.

## SARS‐CoV‐2 virology

**Fig. 1:**
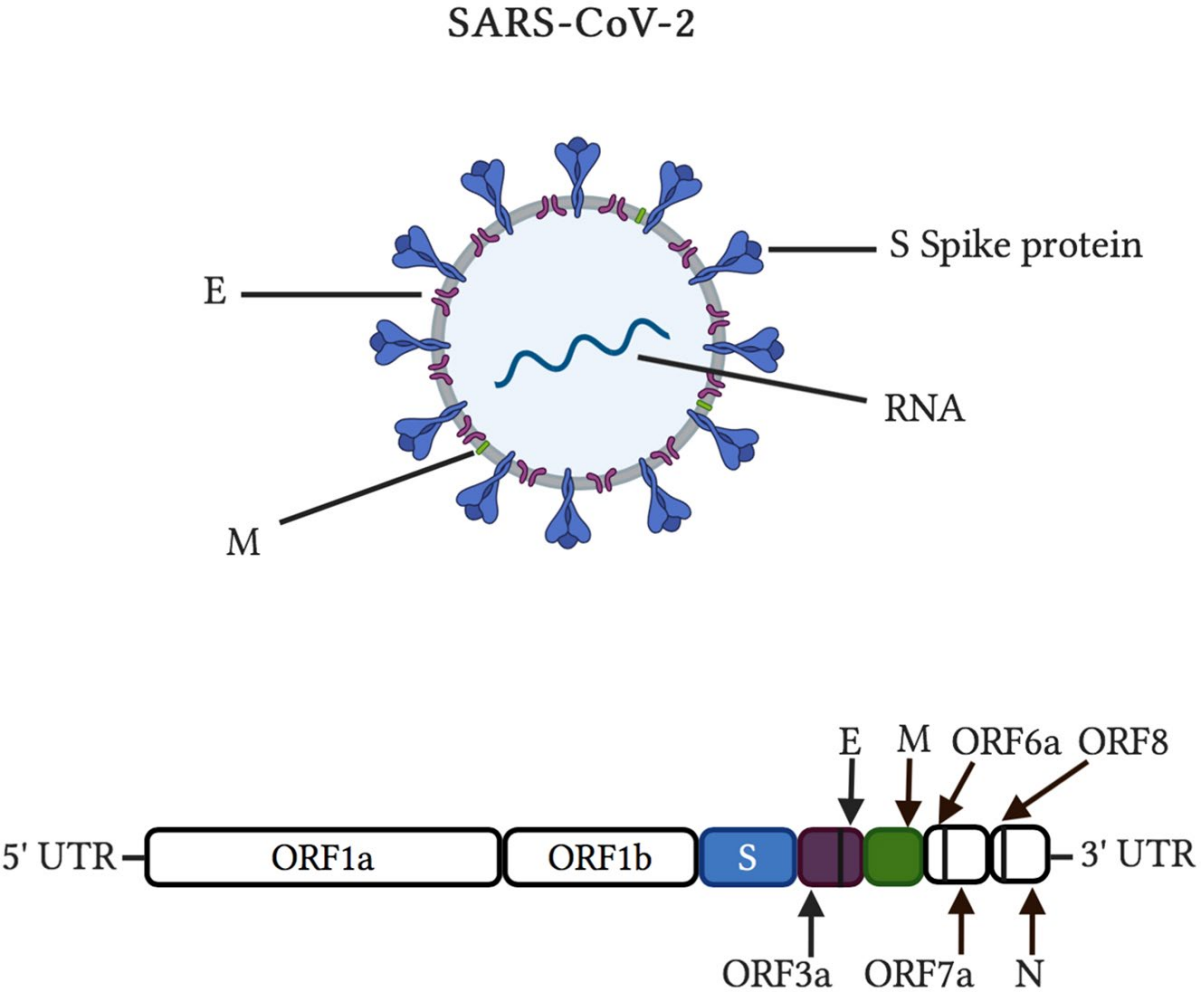
SARS‐CoV‐2 virus. The SARS‐CoV‐2 encodes 11 ORF, ORF1a and ORF1b are polyproteins that are cleaved into multiple individual proteins. The spike (S) protein is the major antigenic determinant, coat is made of spike (S), membrane (M) and envelope (E) proteins. The RNA in encapsulated with the nucleocapsid (N) protein). Created with Biorender.com

Coronaviruses are unusually large enveloped RNA viruses, with a large positive‐sense, single‐stranded RNA genome. The integrity of this lengthy genome is maintained by a proof‐reading replicase. The SARS‐CoV‐2 genome encodes 11 open reading frames (ORF), many of which have unknown functions (Fig. 1). ORF1a and ORF1b both encode polyproteins, which are cleaved into multiple non‐structural proteins. ORF4 encodes the envelope protein, a viroporin [2], and ORF5 encodes the membrane protein; together, they coordinate viral assembly and release. ORF9 encodes the nucleocapsid (N) protein. ORF2 encodes the spike (S) surface glycoprotein, the viral entry protein and key antigenic determinant, which binds the angiotensin converting enzyme 2 (ACE2) receptor on the host cells. ACE2 is commonly found on type II pneumocyte cells in the airways. SARS‐CoV‐2 has a 10–20 times higher affinity for ACE2 than the related coronavirus SARS‐CoV‐1 [3], which was responsible for the 2002–04 SARS outbreak. SARS‐CoV‐2 is able to bind ACE2 from a wide range of mammalian species [4]. Having bound ACE2, spike protein is cleaved by a host cell surface bound proteinase, either Furin or TMPRSS2, enabling entry of the viral capsid. There may be a relationship between the mechanism of viral entry via ACE2 and the pathogenesis of disease.

### Pathology in natural disease

Human coronaviruses can cause both mild (OC43, HKU1, 229E and NL63) and severe (SARS‐CoV‐1, SARS‐CoV‐2 and MERS) disease. For most patients (approximately 80%), SARS‐CoV‐2 causes an asymptomatic infection or mild symptoms [5]. The following signs are associated with a virus positive polymerase chain reaction (PCR) test: fatigue, fever, chills, loss of appetite and persistent cough [6]. A striking feature of infection with SARS‐CoV‐2 is anosmia, a loss of smell and taste, reported in approximately 64% of cases in one study [7]. Whether viral spread to the lower respiratory tract is a precursor for severe disease is unclear; pneumonia with characteristic pulmonary ground glass opacity changes on chest CT scans is common, even in asymptomatic individuals [8]. Blood clotting, respiratory compromise, renal damage and cardiovascular collapse are all features of severe disease. The greatest risk factor for severe COVID‐19 disease is age: the remarkable relationship with age is consistently observed, despite geographic variability in reported case fatality rates [9].

## Immune response to SARS‐CoV‐2

### Protective immunity

While SARS‐CoV‐2 is a new virus and, therefore, the exact correlates of protection are not completely defined, there are precedents from other respiratory infections in general and coronaviruses in particular [10]. There has been discussion that natural immunity to SARS‐CoV‐2 declines quickly; whether this is the case is still unclear. It is our speculation that because vaccines aim to evoke an immune response they could be more immunogenic than the virus itself, which might have mechanisms to dampen immune response: whether this speculation is correct or not is yet to be determined.

#### T cells

The T cell response is important in the control of other respiratory infections, and therefore likely to be important in COVID‐19 [11]. Models of SARS‐CoV‐1 indicate that T cells can be protective. CD4^+^ T cell depletion in mouse models delayed viral clearance and enhanced disease [12]; similarly, T cell transfer resulted in rapid viral clearance and disease amelioration [13, 14]. SARS‐CoV‐1‐specific CD8^+^ T resident memory were protective in a mouse model in the absence of antibody [15]. T cell memory can be long‐lived; SARS‐CoV‐1 T cells were detected 4 years after infection [15, 16]. For SARS‐CoV‐2, T cell responses have been observed to a range of antigens, including S, M, N and other ORFs [17]. SARS‐CoV‐2‐specific T cells have been detected in individuals who had asymptomatic or mild COVID‐19 [18] and SARS‐CoV‐2‐specific T cells have been observed in contacts of infected individuals [19]. Patients suffering from COVID‐19 had fewer T cells than healthy controls [20].

T cells, especially CD4^+^ T cells, can influence the immune response through the production of cytokines, and elevated cytokines have been associated with exacerbated disease [20]. The skewing of the CD4^+^ T cell response is likely to be important. T helper type 1 (Th1) responses are central to the successful control of SARS‐CoV‐1 and MERS‐CoV [21]. Th17 responses have been speculated to be deleterious [22], and increased Th2 cytokines were seen in severe disease [23]. Regulatory T cells are important in the resolution of infection, and were observed to be elevated in COVID‐19 patients [20]. Circulating follicular T helper cells, important in defining recall antibody response to infection, have been observed in a small number of individuals with COVID‐19 [24]. It is not clear whether the ‘cytokine storm’ is a cause or effect of disease; understanding this relationship is critical in monitoring vaccine safety.

#### Antibody response

The humoral response is pivotal in later stages of infection and helps to inhibit subsequent reinfection. Virus‐specific antibodies were detectable in 80–100% of SARS‐CoV‐1 and MERS‐CoV patients 2 weeks after onset of symptoms [25, 26, 27, 28, 29, 30, 31], with delayed antibody responses associated with more severe disease. A number of studies have been performed to try to more clearly understand the antibody response to SARS‐CoV‐2; a systematic review of studies on antibody to coronaviruses [32] observed that antibody was rarely seen in the first 7 days of infection, but rose in the second and third weeks post‐infection. It is unclear whether antibodies correlate with COVID‐19 severity.

Antibodies are likely to be an important part of vaccine‐induced protection. In SARS‐CoV‐1, the antibody response is short‐lived [immunoglobulin (Ig)M and IgA responses last less than 6 months and IgG lasts approximately 1 year]; this is possibly the same for SARS‐CoV‐2 [33]. Human challenge studies using non‐COVID‐19 coronavirus strains suggest that higher antibody levels correlate with protection [32]. These challenge studies have also suggested that reinfection is possible [34], but the dose in challenge studies may be higher than experienced during natural infection. Two recent studies have observed natural reinfections with SARS‐CoV‐2, one asymptomatic [35] and one symptomatic [36], although this is in the context of more than 25 million recorded cases globally, suggesting that it is a rare event. Because of the overlap between SARS‐CoV‐1 and SARS‐CoV‐2 spike proteins, antibodies could be cross‐neutralizing [37]. However, the most potent specific, neutralizing monoclonal antibodies against the receptor binding domain (RBD) of SARS‐CoV‐1 did not bind to the spike protein of SARS‐CoV‐2 [38]. One promising observation is that isolated neutralizing antibodies have minimally mutated VDJ genes, which make inducing them possible with fewer rounds of vaccination [39].

Most attention has focused upon neutralizing IgG antibodies in the serum, but other antibody‐mediated mechanisms may be important in disease pathogenesis. Fragment crystallizable (Fc) and Fc receptor (FcR) interactions can regulate the inflammatory response [40] and the SARS‐CoV‐2 virus–antibody complex could potentially trigger such FcR‐mediated inflammatory responses, causing acute lung injury [41]. The IgA response may be important in determining disease severity of COVID‐19 patients, but remains relatively unexplored so far [42].

## Vaccine‐induced immunopathology

One concern with vaccine development for SARS‐CoV‐2 is that the immune response can cause disease, often in the act of clearing the infection. Understanding vaccine‐induced immunopathology is critically important for all emerging infectious diseases. Vaccines for emerging infections will, by necessity, require a shorter turn‐around from discovery to deployment, and therefore predicting safety early in the process is critical. Vaccine‐induced immunopathology can either present as an acute response to the vaccine itself or as disease enhancement after viral infection.

### Acute immune reaction to vaccination

Vaccines can occasionally induce an acute autoimmune disease. This was observed during the 1976 H1N1 swine flu outbreak, where vaccination in the United States led to an increased risk of Guillain–Barré syndrome (GBS) [43]. The mechanism has not been fully determined, but one suggestion is off‐target antibodies against ganglioside GM1. Off‐target autoimmune effects were also observed during the 2009 H1N1 swine flu pandemic, with narcolepsy observed in a subset of children immunized with a vaccine adjuvanted with AS03 [Pandemrix; GlaxoSmithKline (GSK)]. There was a very tight association with HLA‐DQB1*06:02 [44]. The proposed mechanism is inhibition of the hypocretin signalling pathway. Curiously, another swine flu vaccine made by GSK (Arepanrix; GSK) using the same adjuvant was not associated with narcolepsy [45], suggesting that the side effect was not caused by the adjuvant. The level of viral proteins, specifically nucleoprotein, may have been the problem [46]; anti‐nucleoprotein antibodies have been seen to cross‐react with hypocretin [47]. These acute events are relatively rare; the rate of GBS was 8 per million individuals vaccinated and narcolepsy at approximately 30 per million individuals vaccinated (all in individuals aged less than 20 years) [48]. The delayed effects of vaccines are difficult to predict; post‐licensure monitoring will be critical, especially as the vaccines will potentially have been tested in fewer people during the prelicensure Phases than other licensed products.

### Vaccine‐induced disease enhancement

Disease enhancement following infection of vaccinated individuals has been seen in other viral diseases; for example, measles, respiratory syncytial virus (RSV) and dengue virus. Of children who received formalin‐inactivated measles vaccine and were then subsequently exposed to the wild‐type measles virus, 15–60% developed a severe form of the disease [49], causing the vaccine to be withdrawn in 1967. A similar situation was observed with formalin‐inactivated RSV vaccination (FI‐RSV) in a clinical trial in 1966. The FI‐RSV vaccine induced mainly non‐protective antibodies, and children who were seronegative to the virus prevaccination had enhanced disease and hospitalization compared to the control groups [50]. Vaccine‐enhanced disease has also been observed with the live attenuated tetravalent dengue vaccine (Dengvaxia; Sanofi Pasteur Inc., Swiftwater, PA, USA), specifically in seronegative children [51].

Disease enhancement following vaccination can occur by two main mechanisms: priming for a detrimental T cell response and priming for antibodies that can increase the risk of infection or severe disease.

### T cell immunopathology

The cellular response to vaccination, particularly T cells and eosinophils, and the inflammatory mediators these cells release has been suggested to promote vaccine‐enhanced disease [52, 53, 54]. Whether SARS‐CoV‐2 vaccine platforms will have negative outcomes on infection is currently speculative, and draws upon experience with other respiratory viruses.

One important factor determining the T cell response is antigen selection. Specific epitopes can affect T cell polarization and activation, therefore antigen selection for vaccine applications requires careful consideration [55]. Both the S and N proteins of SARS‐CoV‐1 have epitopes that are recognized by CD4^+^ and CD8^+^ T cells. Some vaccines which used the N protein induced an eosinophilic response associated with vaccine‐enhanced disease [13], and post‐vaccination challenge of animals immunized with SARS‐CoV‐1 N protein induced severe pneumonia [56]. Mismatch of epitopes between vaccine and challenge strain can also lead to T cell enhanced disease due to original antigenic sin, as seen in dengue [57].

The vaccine platform may be critical in determining disease outcome on infection. Immunopathology in animal models has most commonly been linked to inactivated, alum‐adjuvanted vaccines. For example, double inactivation [ultraviolet (UV) and formalin] of SARS‐CoV‐1 enhanced the eosinophilic response from the vaccine, eliciting a proinflammatory pulmonary response and failing to provide complete protection [56]. Enhanced disease was also observed following immunization with a gamma‐irradiated MERS‐CoV vaccine [56]. The mode of inactivation can influence both the quality of antibodies and the polarization of the T cell response to the vaccine. Formalin inactivation in particular has been associated with deleterious Th2 skewing by the addition of carbonyl groups [58], and Th2 skewing has been seen for a formalin‐inactivated vaccine for SARS‐CoV‐1 [59]. Other methods of inactivation have been explored; for example, beta‐propiolactone, UV or gamma radiation, which could prove to be a promising avenue forward for eliciting the correct T cell response [60]. Immunopathology is not restricted to inactivated vaccines. It can occur following immunization with a range of vaccine platforms; for example, it has been seen in animal models of RSV vaccination with both viral vectors and DNA vaccines [61]. Similarly, a range of vaccines against SARS‐CoV‐1 induced Th2‐directed pulmonary immunopathology in mouse models [56]. Age at vaccination may also be an important consideration in immunopathology: the FI‐RSV vaccine was given to infants. Infants have a different immune response to adults, and this may predispose towards a qualitatively different immune memory [62].

### Antibody‐dependent enhancement

The humoral arm of the adaptive immune response can also contribute to disease, called ‘antibody‐dependent enhancement’ (ADE). ADE has been observed with flaviviruses, coronaviruses and some viruses of the *Paramyxoviridae* family [63]. ADE can occur in two ways, either by causing immune complexes or by enhancing infection. Antibodies are bispecific molecules; as such, they can form antigen–antibody complexes. These complexes can cause direct damage when complex deposition in the vasculature leads to complement deposition and vessel damage, as seen after the feline coronavirus infection [64]. Immune complexes can trigger macrophage activation leading to the release of proinflammatory cytokines. Immune complexes have been proposed to have a role in the enhanced disease seen after FI‐RSV immunization [65], and may have a role in SARS‐CoV‐2 [41].

Antibodies can also increase viral disease by enhancing infection; some viruses utilize antibodies to enter target cells. In the case of dengue virus, pre‐existing antibodies for one serotype of the virus can cause enhancement of infection upon subsequent exposure to a new serotype [63]. A number of mechanisms have been proposed: antibody bound to virus could facilitate entry into macrophages through their FcRs [66] and antibody might stabilize viral surface antigen into a mature form [67]. The avidity of the antibody has been suggested as an important factor, with low antibody avidity a risk factor [68]. ADE has been reported in SARS‐CoV‐1 after viral challenge in mice [69], ferrets [70] and macaques [71] using a range of different vaccine strategies. In MERS‐CoV, a neutralizing monoclonal antibody targeting the spike protein promoted viral entry via the Fc receptor [72]. It is not yet known whether antibodies to SARS‐CoV‐2 will enhance disease, but it is something that is being closely monitored [73].

### Models to assess vaccine safety and efficacy

#### Animal models

As coronaviruses have previously been associated with immunopathogenesis, vaccine‐enhanced disease is a potential concern for efficient vaccine design for SARS‐CoV‐2. The use of models can improve understanding [74], potentially predicting correlates of protection or disease. The ideal animal model is permissive to infection with the virus and reproduces the pathology and clinical course observed in humans. Since the SARS‐CoV‐1 outbreak in 2002–04 a range of species, including hamsters, cats, ferrets and non‐human primates, have all been used to study pathogenesis of coronaviruses [75, 76]. Despite productive infection in a wide range of laboratory species, few displayed overt clinical disease.

Several inbred mouse strains have been investigated to model SARS‐CoV‐1, including BALB/c, C57BL/6, RAG1^−/−^ and 129SvEv mice. Although young adult mice infected with varying doses of SARS‐CoV‐1 showed evidence of infection, the inbred strains do not accurately reflect the alveolar damage seen in humans [74]. However, aged mice show signs of clinical disease despite, in many cases, the absence of the lung lesions seen in humans [77], and therefore have been used more extensively than younger mice. Transgenic mice expressing human ACE2 (hACE2) have also been generated; disease severity in transgenic mice largely correlated with the level of hACE2 expression, and when challenged with SARS‐CoV‐1 they developed severe infection and 100% mortality was reached by day 7 [78]. MERS‐CoV appears to be even more challenging to model, with most species resistant to infection, except for some primate species [79] and camelids [80, 81].

The same models are being used for SARS‐CoV‐2. Infection of human ACE2 transgenic mice with SARS‐CoV‐2 led to weight loss and viral RNA was detectable in the lungs, as well as lung pathology [82]. Symptomatic infection and transmission of SARS‐CoV‐2 between animals has been observed in hamsters [83], and asymptomatic infection and transmission of SARS‐CoV‐2 has been observed in ferrets [83]. SARS‐CoV‐2 is also infectious in experimental settings using cats, but not dogs, pigs, chickens or ducks [84]. As with SARS‐CoV‐1, non‐human primates, e.g. rhesus or cynomologous macaques, have been helpful for evaluating immune protection [85].

#### Human challenge

As animal models do not fully recapitulate human disease, alternative strategies may be required. Controlled human infection models (CHIM) are studies in which participants (either vaccinated or not) are intentionally challenged with an infectious organism [86]. CHIM trials of SARS‐CoV‐2 vaccine candidates could be particularly beneficial in vaccine and drug efficacy studies, especially if the community infection rate has declined due to epidemiological interventions [87]. The deliberate exposure of healthy individuals to SARS‐CoV‐2 requires a tight ethical and regulatory framework [88]. The major concerns are that we do not have complete understanding of the long‐term sequelae of SARS‐CoV‐2 infection and there is a lack of rescue therapy to enable the resolution of severe infection, although recent findings suggest that dexamethasone may reduce mortality in severe disease [89] and Remdesivir (Gilead Sciences, Foster City, CA, USA) may improve clinical status [90]. The lack of rescue therapy is not unique to a SARS‐CoV‐2 CHIM. Rhinovirus and RSV CHIM do not have a specific anti‐viral treatment but are self‐resolving, which may also be true for SARS‐CoV‐2 in healthy young adults. There are also challenges associated with the manufacture of a challenge virus stock, which requires a high‐containment [biosafety level III (BSLIII)] laboratory. At the time of writing, no study had been established, although the World Health Organization (WHO) has published guidance [91] and several academic and contract research organizations are investigating the approach [92]. An alternative use of deliberate human infection has been proposed: to infect young, low‐risk individuals to build herd‐immunity, and therefore safeguard the unvaccinated, immunocompromised and immunologically naive [93, 94]. However, this strategy is unattractive because the risk factors for severe disease are not fully understood: ethically there are also questions about infecting groups of individuals for the greater benefit, especially if there is a financial incentive.

## Vaccines against SARS‐CoV‐2

A huge range of vaccine approaches against SARS‐CoV‐2 have been proposed (Table 1). These include traditional approaches – inactivated, live attenuated and protein/adjuvant approaches and more novel, as yet, unlicensed approaches – viral vectors and nucleic acids. This has been a rapidly evolving field and some of the vaccines are more advanced than others. We are focusing upon those that are in clinical trials at the time of writing (Table 2). Several factors need to be considered before any vaccine progresses to widespread usage. First and foremost is vaccine safety and efficacy. Closely linked is the scope for global scale‐up manufacture to produce enough doses to achieve herd immunity.

**Table 1:** Vaccines under consideration by platform and manufacturer/ developer, all stages from pre‐clinical; correct at 1^st^ September 2020. See https://vac‐lshtm.shinyapps.io/ncov_vaccine_landscape/ for updates. Bold are in clinical trials

**Platform (vaccines in development)**	**Developer/ Manufacturer**
RNA (30)	Arcturus, BIOCAD, **BioNTech/Pfizer**, Cansino, CNB‐CSIC, Chimeron Bio, China CDC, Chula VRC, **CureVac**, Elixirgen, Emergex Vaccines, eTheRNA, FBRI, Fudan University/ RNACure Biopharma, GeneOne, Gennova, Greenlight, IDIBAPS, **Imperial College London/ VacEquity Global Health**, Max Planck Institute, **Moderna/ NIAID**, **People’s Liberation Army**, RNAimmune, Rochester clinical research, Selcuk University, Translate Bio/ Sanofi, University of Tokyo, University of Washington, Ziphius
DNA (19)	Aegis, BioNet, Chula VRC, Ege University, Entos Pharmaceuticals, **Genexine**, Immunomic/ PharmaJet, **Inovio**, Karolinska Institute, Mediphage Bioceuticals, National Research Centre (Egypt), **Osaka University/ AnGeS**, Scancell, Statens Serum Institute, Takis, Touchlight Genetics, DIOSynVax/ Cambridge University, UW‐Madison, **Zydus‐Cadila**
Non‐replicating viral vector (29)	Altimmune, Ankara University, Bharat Biotech, **CanSino**, CNB‐CSIC, DZIF, Erciyes University, **Gamaleya Research Institute**, GeoVax, Greffex, ID Pharma, IDIBAPS, ImmunityBio, **Janssen**, AveXis, McMaster University, BIOTEC, National Research Centre (Egypt), **ReiThera**, Stabilitech, Tsinghua University, University of Georgia, University of Manitoba, **University of Oxford/ AstraZeneca**, Valo Therapeutics, Vaxart, Vaxinz.
Replicating Viral vector (21)	Aurobindo, **Beijing Wantai Biological Pharmacy/ Xiamen University**, BIOCAD, DZIF, FBRI, FIOCRUZ, IAVI/Merck, **Institut Pasteur/Themis/Merck/University of Pittsburgh**, Intravacc, Weizmann Institute, KU Leuven, Lancaster University, Sumagen, Tonix Pharma, University of Hong Kong, University of Western Ontario, UW‐Madison, Zydus Cadila.
Inactivated (14)	**Sinopharm/ Beijing institute of biological products/ Wuhan institute of biological products**, Beijing Minhai, **Bharat Biotech**, Erciyes University, **Institute of Medical Biology/ Chinese Academy of Medical Sciences**, KM Biologics, National Research Centre (Egypt), Osaka University, **Research Institute for Biological Safety Problems**, Selcuk University, **Sinovac**, Valneva.
Live Attenuated (4)	Codagenix/Serum Insitute India, Indian Immunologicals Ltd, Abicadem, Meissa
Protein (71)	AdaptVac, **Adimmmune**, AJ Vaccines, Akers Biosciences, **Anhui Zhifei**, AnyGo, Applied Biotechnology Institute, Axon Neuroscience, Baiya Phytopharm, Baylor Colloge, Biological E, BiOMVis, Bogazici, Chulalongkorn, **Clover Biopharm/ GSK**, **Covaxx**, EpiVax, ExpreS2ion, FBRI, Flow Pharma, G+Flas life science, Generex, Heat Biologics, Helix Biogen, iBio, ImmunoPrecise, IMV Inc, InnoMedica, Innovax, **Instituto Finlay**, Intravacc, Izmir Biomedicine, **Kentucky Bioprocessing**, LakePharma Inc, Liaoning Chengda, Lomonosov Moscow State University, Max Planck Institute, **Medigen**, MIGAL, MOGAM, Mynvax, Shionogi, National Research Centre (Egypt), Neovii, Oncogen, BIKEN, PDS Biotech, Quadram Institute, Research Institute for Biological Safety Problems, **Sanofi/ GSK**, Sichuan University, SK Biosciences, Soligenix, St Petersburg Scientific research institute of vaccines and sera, University of Alberta, UCSD, University of Pittsburgh, **University of Queensland/ CSL/ Sequirus**, CONICET, University of Virginia, Vabiotech, Vaxil Bio, **Vaxine Pty**, Versatope, VIDO‐InterVac, Walter Reed Army Institute of Research, Yisheng
VLP/ Nanoparticle (13)	ARTES, Bezmialem Vakif, Doherty Institute, Imophoron, IrsiCaixa, Mahidol University, **Medicago/ GSK**, Middle East Technical University, **Novavax**, Navvarabiomed, OSIVAX, Saiba, University of Sao Paolo, VBI Vaccines
Cell based (4)	**Shenzhen Geno‐Immune Medical Institute**, Henan Provincial centre for Disease Control and Prevention, **Avita Biomed**
Bacterial Vector (3)	**Symvivo**, UCLA, Versatope

**Table 2:** **Vaccines in clinical trials**: Correct at 1^st^ September 2020. See https://vac‐lshtm.shinyapps.io/ncov_vaccine_landscape/ for updates. Published data included where found: this may be an incomplete record

Company/Organisation developing	Platform	Manufacturer/Sponsor Location	What antigen	Previous experience for other pathogens	Funding source (where public)	Vaccine available prediction	Clinical Trial stage	Reported Results
Arcturus	self‐amplifying RNA (saRNA)/LNP	Singapore	Pre‐fusion Spike	Influenza, Gene Therapy			Phase I NCT04480957	On company website: https://arcturusrx.com/
BioNTech/Pfizer/Forsun	Modified nucleoside mRNA LNP formulation	Germany	Spike Receptor binding domain (RBD)	Phase I cancer vaccine	Pfizer contract Forsun for China	Early 2021	Phase I (China) NCT04523571 Phase I (China) ChiCTR2000034825 Phase I/II (Germany, USA) NCT04380701 Phase II/III (USA, Argentina, Brazil) NCT04368728	Pre‐clinical (mice) [197] Phase I trials [198, 199, 200]
CureVac	mRNA	Germany	Spike	Rabies, Lassa, Yellow Fever, RSV, Influenza	BMGF, CEPI, EU	Expects clinical tests by June	Phase I NCT04449276	On company website: https://www.curevac.com/en/
Imperial College London/VacEquity Global Health	saRNA/LNP	UK	Stabilised Spike	EBOV; LASV, MARV, Inf (H7N9), RABV	UK government (MRC and NIHR)	June 2021	Phase I/II ISRCTN 17072692	Pre‐clinical (mice) [103]
Moderna	mRNA	USA	Stabilised Spike	MERS‐CoV, CMV, Zika, RSV, PIV3, RSV, Influenza	CEPI	June 2021 Emergency for HCW 2020	Phase I NCT04283461 Phase II NCT04405076 Phase III NCT04470427	Pre‐clinical (mice) [195] Phase I trial [196]
People’s Liberation Army/Walvax	mRNA	China	Spike RBD	Meningococcus, HiB, *Streptococcus pneumoniae*			Phase 1 ChiCTR2000034112	None published
Genexine	DNA	Korea	Spike	HPV			Phase I NCT04445389	None published
INOVIO	DNA	USA	Spike	MERS‐CoV, HPV, HIV, Ebola, Lassa	BMGF, CEPI, DoD		Phase I (USA) NCT04336410 Phase I/II (Korea) NCT04447781	Pre‐clinical (mice) [185] Pre‐clinical (NHP) [186] Phase I trial on company website https://www.inovio.com/
Osaka University/AnGes	DNA	Japan	Spike	Gene therapy	Unknown		Phase I NCT04463472	None published
Zydus Cadila	DNA	India	Spike	Rabies, Flu, MMR, Tetanus		Early 2021	Phase I CTRI/2020/07/026352	On company website: https://zyduscadila.com/research
CanSino Beijing Institute of Biotechnology	Adenovirus Type 5 Vector	China	Spike	Ebola	Unknown	2021	Phase I ChiCTR2000030906 Phase II (China) NCT04341389 Phase I/II (Canada) NCT04398147	Phase I [166] Phase II [167]
Gamaleya Research Institute	Adenovirus prime boost (Ad26 and then Ad5)	Russia	Spike			2021 Jan ‘Sputnik V’	Phase I NCT04436471 Phase I (lyophilised) NCT04437875 Phase III NCT04530396	Phase I [171]
Janssen	Ad26 adenovirus vector	USA, Belgium	Spike	Ebola (Ad26.ZEBOV)	N/A		Phase I/II (USA/Belgium) NCT04436276 Phase I (Japan) NCT04509947 Phase III (Multi‐site) NCT04505722 (not yet started)	Pre‐clinical (NHP) [170]
University of Oxford/AstraZeneca	Adenovirus: ChAdOx1 nCov‐19/AZD1222	UK	Spike	MERS, influenza, TB, Chikungunya, Zika, MenB, plague	UK Government, CEPI, US Government	Early 2021	Phase I/II (UK) NCT04324606 Phase I/II (South Africa +/‐ HIV) NCT04444674 Phase II/III (UK) NCT04400838 Phase II/III (Brazil) ISRCTN89951424 Phase III (USA) NCT04516746	Pre‐clinical (mice/pigs) [162] Pre‐clinical (NHP) [163] Phase I/II [164]
Reithera	Simian Adenovirus	Italy	Spike	Gene delivery/Vectored vaccines	Italian Government		Phase I 2020‐002835‐31	None published
Institut Pasteur/Themis/Merck/University of Pittsburgh	Live attenuated recombinant measles vector	Austria/USA	Modified Spike	Chikungunya virus	CEPI		Phase I NCT04497298 NCT04498247	None published
Beijing Wantai Biological Pharmacy/Xiamen University	Influenza virus vector	China		Hepatitis E vaccine			Phase I ChiCTR2000037782	None Published
Beijing institute of biological products/Wuhan institute of biological products/Sinopharm	Beta‐propiolactone inactivated virus	China	Whole virus			Dec 2020	Phase I/II (China) ChiCTR2000031809 Phase III (UAE) ChiCTR2000034780 Phase III (UAE, Bahrain) NCT04510207	Phase I/II [150]
Bharat Biotech	Inactivated virus	India	Whole Virus		Indian Council of Medical Research		Phase I/II (India) NCT04471519	None Published
Institute of Medical Biology, Chinese Academy of Medical Sciences	Inactivated virus	China	Whole Virus				Phase I/II NCT04412538 Phase I/II (Over 60s) NCT04470609	None Published
Research Institute for Biological Safety Problems/National Scientific Center for Phthisiopulmonology of the Republic of Kazakhstan	Inactivated virus	Kazakhstan					Phase I NCT04530357	None Published
Sinovac	Beta‐propiolactone Inactivated Alum Adjuvant	China	Whole inactivated virus adjuvanted Alum or CpG	Hand‐Foot and Mouth, Hepatitis A, Influenza	Ministry of science and technology, China	Late November 2020	Phase I/II (China) NCT04383574 Phase I/II (China) NCT04352608 Phase III (Brazil) NCT04456595 Phase III (Indonesia) NCT04508075	Phase I [149]
Adimmune	Protein (Baculovirus derived) +Alum adjuvant	Taiwan	Spike Receptor binding domain (RBD)	EV71, Influenza, Japanese Encephalitis Virus			Phase I NCT04522089	None published
Anhui Zhifei	Protein	China	RBD Dimer				Phase I NCT04445194 Phase II NCT04466085	None published
Clover Australia and GSK	Protein subunit S‐Trimer AS03 adjuvant	Australia	Trimeric SARS‐CoV‐2 S protein subunit AS03 Adjuvant	Influenza	CEPI funding for phase I, adjuvant provided by GSK		Phase I NCT04405908	None published
Covaxx/University of Nebraska Medical Center	Multi‐epitope peptide	Taiwan/USA	RBD peptide plus CTL pool from M, S2 and N				Phase I NCT04545749	None published`
Vector Institute	Peptide + Adjuvant (Alum)	Russia	Multiple epitopes				Phase I NCT04527575	None published
Instituto Finlay de Vacunas	Protein + Adjuvant	Cuba	Spike RBD				Phase I IFV/COR/04	None published
Kentucky Bioprocessing	Protein + (Plant derived)	USA	Spike	Influenza			Phase I NCT04473690	None published
Medigen	Protein + CPG + Alum	USA	Spike				Phase I NCT04487210	None published
Sanofi/GSK	Protein + Adjuvant (AS03? + other)	France/USA	Spike	Multiple		Early 2021	Phase I/II NCT04537208	None published
University of Queensland/CSL	Protein + adjuvant (MF59)	Australia	Clamped Spike protein	Influenza, RSV	Australian Government CEPI	Early 2021	Phase I NCT04495933	None published
Vaxine PTY	Protein + Adjuvant (Advax)	Australia	Spike	Influenza, JEV, West Nile			Phase I NCT04453852	None published
West China Hospital/Sichuan University	Protein (insect cell derived)	China	RBD				Phase I NCT04530656	None published
Novavax	Recombinant nanoparticle vaccine (NVX‐CoV2373) Matrix M adjuvant	USA	Spike	Previous vaccine phase I work for SARS, MERS and Ebola	CEPI	June 2021	Phase I NCT04368988	Pre‐clinical (Mice/NHP) [129] Phase I [128]
Medicago	Protein VLP (Plant derived) + CpG + AS03	Canadia	Spike				Phase I NCT04450004	None published
Avita	Dendritic cell vaccine +GMCSF	USA					Phase I NCT04386252	None published
Shenzhen Geno‐Immune Medical Institute	Lentiviral transfected artificial APC (and DC)	China	S, M, E, N and P proteins			2023/24	Phase I NCT04276896 Phase I NCT04299724	None published
Symvivo	*Bifidobacterium longum* vector, delivered orally	Canada	Spike				Phase I NCT04334980	None published

### Possible antigens

#### The spike (S) protein

Before looking at the platforms being developed, the antigen needs to be considered. Based on experience with SARS‐CoV‐1, most vaccines target the SARS‐CoV‐2 spike protein. Within the spike, the receptor binding domain (RBD) responsible for binding to and entering host cells is the primary target of neutralizing antibodies [95], and some vaccines only include this region. However, a recent study that isolated monoclonal antibodies found that most of them targeted areas outside the RBD [39]. An important consideration is the correct folding of the protein, both during production and when the vaccine is in storage prior to deployment. The coronavirus spike is a type 1 fusion protein and is metastable, undergoing an irreversible conformational change to enable membrane fusion [3, 96, 97]. This may affect the ability of the antigen to induce neutralizing antibodies. A similar effect has been seen with the RSV fusion (F) glycoprotein. Antibodies specific to prefusion F (pre‐F) have better neutralizing capacity than post‐fusion F‐specific antibodies [98, 99, 100, 101]: stabilization of the pre‐F form can lead to better responses. Based on this, prefusion SARS‐CoV‐2 spike protein could elicit a more potent immune response and stabilized SARS‐CoV‐2 spike proteins have been generated with stabilizing proline mutations in the S2 domain [3, 102, 103].

#### The nucleocapsid (N) protein

Coronavirus nucleocapsid (N) is also immunogenic: antibodies against the SARS‐CoV‐1 N protein are abundant and longer‐lived than those against the S protein in recovered patients [104]. Interestingly, in model systems of SARS‐CoV‐1, immunization with the N protein is associated with vaccine‐enhanced disease [105, 106]. It is not known whether the N protein is a potential protective immunogen for SARS‐CoV‐2, although vaccine approaches that use whole virus – either inactivated virus or live attenuated approaches – will potentially include N protein. The N protein can be a useful diagnostic for infection during Phase III trials of S protein‐based vaccines.

#### T cell epitopes

While the emphasis has been on the generation of neutralizing antibodies, targeting T cell epitopes may provide additional protection [11]. In other respiratory viruses, for example RSV, T cell only strategies can enhance disease [61] and T cells can be deleterious in dengue [57], although less evidence of this has been seen with influenza vaccines [107]. It is not yet clear whether SARS‐CoV‐2 behaves more like RSV or influenza. Drawing on information about SARS‐CoV‐1 and MERS‐CoV and using bioinformatics, potential immunogenic epitopes in the SARS‐CoV‐2 proteome have been predicted. A total of 781 human leucocyte antigen (HLA) class I and 418 HLA class II epitopes common between SARS‐CoV‐1 and SARS‐CoV‐2 were found [108]. T cell responses against the structural proteins of SARS‐CoV‐1 were found to be more immunogenic than non‐structural proteins [13].

## Platforms

**Fig. 2:**
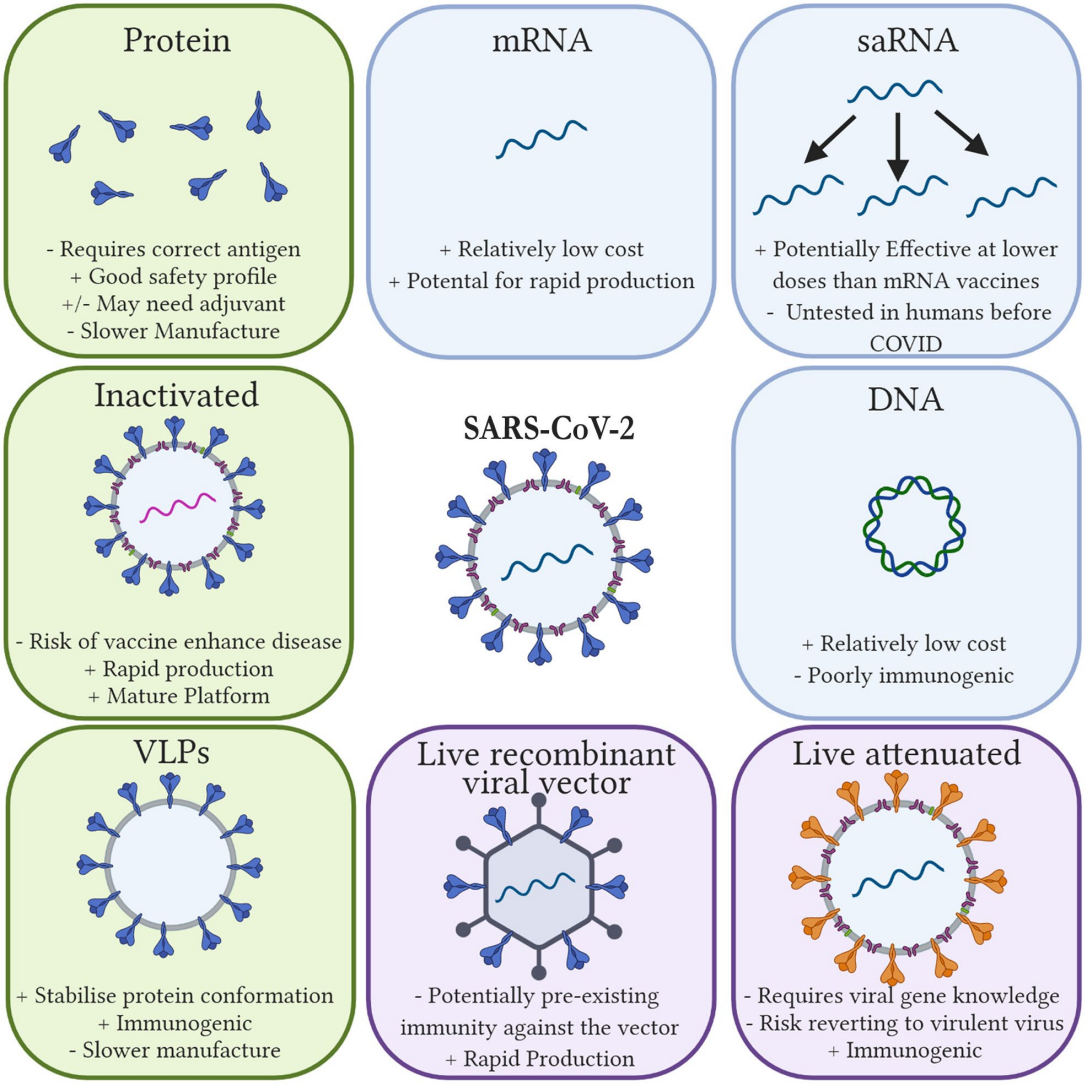
Vaccine platforms; Over 200 different vaccines are in development. They loosely group into protein, inactivated, VLP, viral vector, mRNA, self‐amplifying RNA, DNA and live attenuated vaccines. Created with Biorender.com

A wide range of different platforms have been developed, which can be loosely grouped as proteins, inactivated virus, vectored vaccines, live attenuated and nucleic acid (Fig. 2). This is clearly a fast‐moving space and the following is based on data accessed in September 2020; an updated website is available at https://vac‐lshtm.shinyapps.io/ncov_vaccine_landscape/ and the WHO has a vaccine tracker [109]. As many of the vaccines under development are produced by commercial organizations, peer‐reviewed publications concerning their development and efficacy are limited, as such some information has been taken from press releases which may be less robust in their scrutiny. Published results from clinical trials are summarized in Table 3.

**Table 3:** Data from Published Phase I studies. Data from peer‐reviewed journals or pre‐prints. Data published on company websites not included

Vaccine manufacturer	Safety	Immunogenicity	Reference
University of Oxford/AstraZeneca	Mild/Moderate injection site pain Mild‐Severe systemic adverse reactions including chills, fatigue, malaise and headache, peaking day one, reduced by paracetamol.	Seroconversion with neutralising antibodies, (91% after one dose, 100% after two doses). IFNγ ELISPOT responses detected,.	[165]
Wuhan Institute of Biological Products/Sinopharm	Local reactions in 25% of highest dose (Phase I) Adverse reactions in 6‐19% (Phase II)	Phase I: 95‐100% seroconversion (ELISA and neutralisation). Phase II: 85‐100% seroconversion	[149]
BioNTech/Pfizer	Dose 1: Local reactions in 100% of 30µg (mild‐moderate) and 100µg groups (mild‐severe) Systemic reactions in up to 80% of 100µg (mild‐severe). Dose 2: systemic reactions in 100% of 30µg group (mild‐severe) Similar results seen in second study with same construct (BNT162b1) Comparative study with alternate construct (BNT162b2) showed lower reactogenicity	Seroconversion with neutralising antibodies and ELISA binding Higher response in higher dose group Neutralising antibody increased on booster in 10µg and 30µg groups. Similar results seen in second study with same construct Comparative study with alternative construct had equivalent immunogenicity	[198, 199, 200]
CanSino Biological Inc./Beijing Institute of Biotechnology	Phase I: Local reactions in 54% (mild or moderate) Systemic adverse events in 46 % (mild‐severe) No effect of dose size on effects. Phase II: Adverse reactions in 72%, more severe events in larger dose group (9%). Including injection site pain (56%) and fever (32% in high dose) Adverse effects lower in individuals with pre‐existing anti‐Ad5 antibodies	Phase I: Seroconversion (ELISA binding) 44‐61% after 1 dose, 97‐100% after 2 doses. Seroconversion (Neutralising) 28‐42% after 1 dose, 50‐75% after 2 doses. IFNγ ELISPOT responses detected. Phase II: Seroconversion (Neutralising) 59‐61% after 2 doses. IFNγ ELISPOT responses detected after 2 doses.	[166, 167]
Moderna/NIAID	Systemic adverse events – mild/moderate after first dose, increasing with µg RNA administered. More adverse effects on second dose. All of 250 µg group reported systemic adverse effects after second dose, 21% were severe.	100% seroconversion by after second dose by ELISA and neutralisation. Increase in response from 25 µg to 100µg dose, rough equivalence between 100µg and 250µg dose. Antigen specific T cells detectable, greater in 100µg group than 25µg.	[196]
Sinovac	Low rate of adverse effects – no different to placebo	90% seroconversion reported by ELISA and neutralisation Slight reduction in titre in older groups.	[148]
Gamaleya Research Institute	Mild‐moderate systemic adverse effects (mild fever in 95% of volunteers) for liquid formulation, less for lyophilised. Mild local adverse effects (injection site pain)	100% seroconversion reported by ELISA and neutralisation. Anti‐vector antibodies observed but did not correlate with sero‐conversion or anti‐RBD titre. Antigen specific IFNγ T ELISPOT responses.	[171]
Novavax	Mostly mild systemic adverse effects, some moderate‐severe, increased severity on second dose; headache and fatigue most common. Mild‐moderate local adverse effects (Tenderness and site pain). Increased adverse effects with adjuvant.	100% seroconversion by ELISA in groups with adjuvant, no difference in antibody response between high (25µg) and low (5µg) groups. Boosting effect observed by 2 shots. Neutralisation titres much greater in adjuvanted groups, but only 100% after boost. Subset had T cell responses analysed.	[128]

## Protein vaccines

As with other pathogens, recombinantly produced viral surface proteins can safely be used as vaccines for COVID‐19. Although protein vaccines have a good safety profile they can have low levels of immunogenicity, which means that many require adjuvants to improve their efficacy. While bacterial protein vaccines can be made through the purification of whole pathogen preparations, viral subunit vaccines necessitate recombinant genetic engineering. The genes encoding the chosen antigens are cloned or synthesized, expressed and purified using a variety of expression systems, including insect, bacterial, yeast and mammalian cells [110]. Bacterial expression systems are often used because they have high levels of expression and are easy to scale‐up, with fermenter repurposing relatively easy. However, for viral antigens, where post‐translational modification can be important, the use of insect cells or mammalian cells may be preferential [111, 112].

### Protein vaccines for other coronaviruses

Several protein subunit vaccination approaches were under development for SARS‐CoV‐1 and MERS‐CoV [113, 114, 115]. A subunit vaccine made up of SARS‐CoV‐1 spike protein fragments, expressed in *Escherichia coli*, induced neutralizing antibodies in rabbits [116]. Neutralizing antibodies were induced in mice after immunization with transgenic plants [117] or mammalian expressed [118] recombinant SARS‐CoV‐1 spike protein. Most MERS‐CoV subunit vaccines use mammalian cell‐expressed spike protein [113, 114, 115, 119].

### Protein vaccines in development for SARS‐CoV‐2

Several SARS‐CoV‐2 protein vaccines are in development; eight candidates are in clinical trials, but no data are yet available from these trials. Two of the earliest to be announced are from Clover Biopharmaceuticals and the University of Queensland. Clover Biopharmaceuticals has used ‘Trimer‐Tag’ technology to make a mammalian cell‐expressed, spike protein subunit trimer vaccine [120]. This antigen can be recognized by antibodies in the sera of people who have recovered from SARS‐CoV‐2 [121]. The vaccine will be given in conjunction with GSK’s adjuvant AS03 or cytosine–phosphate–guanosine (CpG)/alum during the Phase I trial (NCT04405908). The University of Queensland, funded by the Coalition for Epidemic Preparedness Innovations (CEPI) has developed a recombinant subunit vaccine using spike protein that has been ‘locked’ in prefusion conformation using the molecular clamp technique [122]. This is currently being tested with MF59 (NCT04495933).

Sanofi are developing a protein subunit vaccine against SARS‐CoV‐2, expressed using a baculovirus platform, funded by the US Biomedical Advanced Research and Development Authority (BARDA). This has been reported to be delivered in conjunction with AS03 from GSK [122, 123] or potentially one other adjuvant which has not been revealed. Phase I clinical trials were initiated on 3 September 2020 (NCT04537208), with an aim to make the vaccine available in early 2021 [122].

Other protein candidates in clinical trials (Table 2) are from Adimmune (baculovirus‐derived, alum adjuvanted), Anhui Zhifei (RBD only), Instituto Finlay de Vacunas (RBD), Kentucky Bioprocessing (tobacco‐derived protein), Medigen (alum/CpG adjuvanted) and Vaxxine (Advax adjuvanted). Differences in cost of manufacturing, location of the manufacturer and impact of the adjuvant will determine which candidates progress beyond clinical trials.

## Nanoparticles and virus‐like particles

Virus‐like particles (VLPs) are a subset of protein vaccines which are artificially produced nanoparticles that resemble viruses. Rather than an individual protein, VLPs are made up of some or all of the proteins that form the viral capsid [124]. They have some similarities to live attenuated or inactivated vaccines, and can produce strong cellular and humoral immune responses with no risk of reversion, because they contain none of the genetic material of the virus. They are used for a wide range of viruses, including HPV, and a preclinical SARS‐CoV‐1 VLP has been tested [125]. VLP Nanoparticles are self‐assembling protein particles, not necessarily derived from the virus capsid proteins.

Novavax, funded by CEPI and US Operation Warp Speed, have developed a recombinant nanoparticle vaccine (NVX‐CoV2373) that displays the SARS‐CoV‐2 spike protein [122, 126]. This is produced using engineered baculovirus to infect Sf9 insect cells [127]. For the clinical trial with NVX‐CoV2373 Novavax are using their own saponin‐based Matrix‐M adjuvant (NCT04368988), the data from which have recently been published [128]. The vaccine was immunogenic, but required the addition of adjuvant to achieve 100% seroconversion; two doses were required for neutralising antibody in all individuals. Immunized animal models develop spike protein‐specific antibodies that prevent the attachment of the spike protein to host cell ACE‐2 receptors and also neutralize the wild‐type virus [129]. Another company (Medicago) are using a plant‐based system, *Nicotiana benthamiana*, to produce a VLP [130] which is currently in clinical trial in combination with CpG or AS03 adjuvant (NCT04450004).

Other groups at the preclinical stage include Saiba AG, based in Switzerland, who are using a cucumber mosaic virus VLP that is bound to SARS‐CoV‐2 RBD, which induced neutralizing antibody in mice [131].

## Peptide vaccines

Peptide vaccination is based upon the concept that, as induction of T cell responses can be achieved using a fraction of the entire protein [132, 133], only the minimal immunogenic peptide sequence needs to be included. By selecting conserved epitopes, peptide vaccines can potentially induce broad‐spectrum immunity against multiple strains of a given pathogen [134, 135]. Peptides are easier to produce than whole protein antigens, as they can be produced synthetically and do not require folding into a tertiary structure. However, peptide vaccines are often weakly immunogenic. This is due to several factors, including the relatively small size of the peptide and differences in MHC processing; they therefore may require carrier proteins or adjuvants [136, 137]. Several groups are exploring the use of multi‐epitope peptide vaccines against SARS‐CoV‐2; following bioinformatic and immune‐informatic‐based predictions of immunogenic epitopes [138, 139, 140, 141], the studies are focusing upon T rather than B cell epitopes. OSE Immunotherapeutics have used a multi‐epitope peptide approach to induce T cell responses in mice [142]. Covaxx and the University of Nebraska Medical center have recently registered a Phase I clinical trial for a multi‐epitope peptide vaccine (NCT04545749) as has the Vector Institute (NCT04527575) currently in clinical trial.

## Artificial antigen‐presenting cells

Artificial antigen‐presenting cells (aAPC) are immunotherapeutic agents that can stimulate antigen‐specific T cell responses [143] They have been widely explored for cancer vaccines and have also been proposed for infectious disease vaccines. In the case of SARS‐CoV‐2, aAPCs are transfected with a lentivirus encoding the structural and protease proteins. The cells are then administered via subcutaneous injection [144]. The Shenzhen Geno‐Immune Medical Institute in China are undertaking an ongoing Phase I clinical trial with an aAPC approach (NCT04299724) and a modified dendritic cell platform (NCT04276896). Aivita Biomedical Inc. are following a similar platform (NCT04386252). Due to the need to isolate and purify cells and maintain them at GMP quality, this approach seems impractical for mass vaccination campaigns.

## Inactivated vaccines

Isolating and then inactivating a virus, historically with formaldehyde, is one of the oldest methods of viral vaccination. Inactivation of viruses has been effective for a range of different viruses. However, there have been major safety concerns relating to SARS‐CoV‐1 and MERS‐CoV‐inactivated vaccines, reminiscent of FI‐RSV, and these concerns are also valid for SARS‐CoV‐2. Lung pathology of vaccinated animals on virus challenge has been seen for both a gamma‐irradiated MERS‐CoV vaccine [56] and a UV irradiation‐inactivated SARS‐CoV‐1 vaccine [145]. The choice of both the adjuvant and the inactivating agent is important in shaping the immune response. For example, a formaldehyde inactivated MERS‐CoV vaccine adjuvanted with alum and CpG demonstrated enhanced protection without inducing eosinophil‐mediated vaccine‐related pathology [146].

### Inactivated viral vaccines in development for SARS‐CoV‐2

There are four inactivated vaccine candidates in clinical trials. Sinovac Biotech are using a platform previously developed for SARS‐CoV‐1 [147]. The virus is grown in Vero cells and inactivated with beta‐propiolactone. The inactivated vaccine was safe and immunogenic in rhesus macaques and offered complete protection against SARS‐CoV‐2 challenge, where no virus was detected in the pharynx or lungs [148]. Two different versions of this inactivated vaccine have been developed, adjuvanted with either alum or CpG108. This vaccine has completed a Phase II human trial in 600 healthy adults aged 18–59 years (NCT04352608), with 90% seroconversion observed after the second dose of vaccine and some neutralizing antibody detected [149]. It is interesting to note that the production method for the virus was changed between Phases I and II trials, and this may have increased immunogenicity. The vaccine has entered Phase III clinical trials in Brazil (NCT04456595) and Indonesia (NCT04508075).

Sinopharm, working with both the Beijing Institute of Biological Products and the Wuhan Institute of Biological Products, have also developed an inactivated vaccine. This vaccine has now been tested in a Phases I/II clinical trial (ChiCTR2000031809). No serious adverse effects were observed, and more than 95% of individuals seroconverted with detectable neutralizing antibody in the two different trials [150]. The antibody was mainly observed after the second dose.

Two other organizations, Bharat Biotech (India) and the Institute of Medical Biology/Chinese Academy of Medical Sciences, are running clinical trials of inactivated vaccines, but these are ongoing with no published data as yet. Valneva, based in Scotland, have just expanded their BSL3 manufacturing capacity and have signed a deal with the UK government for 100 million doses of a formaldehyde‐inactivated vaccine adjuvanted with CpG, based on their Japanese encephalitis virus vaccine [151].

## Live vaccines

The use of a live virus to prevent infection is the oldest vaccine approach. The original vaccine, cowpox, used exactly this approach to prevent smallpox. We are grouping two approaches under live viral vaccine platforms: attenuation of the virus or the use of a viral vector to deliver transgenes.

### Live attenuated vaccines

Live attenuated vaccines closely resemble natural infection. As a result, they are often immunogenic with a single administration without an adjuvant [152]. One consideration is balancing attenuation and replication – over‐attenuated vaccines may not replicate enough to be immunogenic, and this balance can vary between different individuals, especially the very young or immunocompromised. Historically, serial passage for attenuating mutations has been used; for example, live attenuated influenza vaccine (LAIV) is cold‐adapted, restricting it to the upper airway. This method requires time and extensive testing: the yellow fever vaccine YF17D was passaged more than 200 times. Alternatively, attenuated viruses can be generated by reverse genetics [153], introducing site‐directed mutations into genes associated with virulence. The E protein has been targeted for both SARS‐CoV‐1 and MERS‐CoV [154, 155]. However, this method requires the identification of genes that would attenuate viral replication and the mutation(s) inserted to be phenotypically stable [153]. A novel method of codon‐pair de‐optimization has been developed. The codon de‐optimized virus is chemically synthesized to retain 100% amino acid sequence identical to the parent virus, but to contain an increased number of CpG and UpA RNA dinucleotides to up‐regulate host responses. Codon‐pair de‐optimization has been used for attenuating RSV [156]. Codagenix and the Serum Institute of India are developing a live attenuated SARS‐CoV‐2 vaccine, using codon de‐optimization technology, building on previous experience with RSV and influenza [157].

### Vectored vaccines

In vectored vaccines, the antigenic gene of interest is expressed from another micro‐organism, either virus or bacteria. Adenovirus, VSV and modified vaccinia virus Ankara (MVA) are some of the common viral vectors used [158]. The vectors can either be replication‐deficient, delivering a gene cargo but not growing themselves, or replication‐competent, reproducing in the immunization site. The different platforms may alter the reactogenicity and immunogenicity of the vaccine.

A recombinant MVA expressing the SARS‐CoV‐1 S protein delivered via intranasal or intramuscular routes induced protective immunity in mice [159]. An adenovirus vaccine against MERS‐CoV offered complete protection against challenge in mice [152]. As pre‐existing immunity against human adenovirus is widespread and can hamper its clinical application as a vector [158], a chimpanzee adenovirus can be used. A recombinant chimpanzee adenovirus (ChAdOx1) encoding the S protein, known as MERS001, was immunogenic in mice and safe in Phase I clinical trials in humans [160].

## Non‐replicating vectored vaccines in development for SARS‐CoV‐2

Five non‐replicating viral vectored vaccines are currently in clinical trials all based around adenoviral vectors. Replication‐deficient adenoviral vectors lack the E1A and E1B genes; these are the early genes which are essential for reproduction of the virus [161], and deliver the antigen gene without replicating in the vaccinated individual.

Building on experience with MERS‐CoV, the University of Oxford are developing a chimpanzee adenovirus vaccine vector expressing the wild‐type S protein (ChAdOx1 nCov‐19, also known as AZD1222). The AZD1222 vaccine was immunogenic in mice and pigs [162]. In rhesus macaques it reduced viral load and pneumonia after challenge with SARS‐CoV‐2 [163, 164]. The AZD1222 vaccine entered Phase I clinical trial on April 23 2020 in 543 volunteers aged 18–55 years (NCT04324606). In this study, there were local and systemic reactions to the vaccine, controlled by paracetamol, but no severe adverse effects. The vaccine was immunogenic, with 91% participants having neutralizing antibody after one dose and 100% after two doses. Interferon (IFN)‐γ‐producing T cells were also detectable [165]. In partnership with AstraZeneca, this vaccine received a further $1.2 billion from BARDA towards its global development, manufacturing and distribution. The vaccine has now progressed into Phases II/III trials in the United Kingdom (NCT04444674), Brazil (ISRCTN89951424) and the United States (NCT04516746).

Cansino Biologics (China) are developing a human Ad5‐vectored vaccine. In Phase I trials (ChiCTR2000030906), the Ad5 vectored COVID‐19 vaccine was tolerable and immunogenic at 28 days post‐vaccination [166]. Both humoral responses and specific T cell responses were observed in healthy individuals 28 days after vaccination. Transient and self‐limiting adverse events such as severe fever, fatigue and muscle pain were reported in the high vaccine dose group. Similar results were reported after the Phase II trial [167]. The vaccine is now in a Phases I/II trial in Canada (NCT04398147).

Janssen (part of J and J) are using an experimental, replication incompetent adenovirus vector (AdVac^®^) in their PER.C6^®^ cell line technology [168]. This platform has been used for Zika, RSV and HIV vaccine candidates. An Ebola vaccine (Ad26.ZEBOV) using the same platform has been proven safe and immunogenic, and has been used as part of efforts to contain Democratic Republic of the Congo (DRC) Ebola outbreaks [169]. The vaccine has been seen to be protective against SARS‐CoV‐2 challenge in rhesus macaques [170] and is in Phase I trials in the United States, Belgium (NCT04436276) and Japan (NCT04509947).

One other vectored vaccine that has received a great deal of press attention is from the Gamaleya Research Institute, which has been given the tradename Sputnik V. This vaccine uses two different adenovirus vectors, Ad26 and Ad5. To date, two clinical trials have been registered giving individually or as a prime‐boost, either as a solution (NCT04436471) or lyophilized formulation (NCT04437875) in a total of 75 people. The trial recorded mild–moderate systemic effects and mild local effects, including injection site pain; 100% seroconversion rate by binding enzyme‐linked immunosorbent assay (ELISA) was observed. Interestingly, there was also some anti‐vector antibody detected after immunization [171]. The registration of this vaccine is presumably subject to larger efficacy trials, with a Phase III trial registered in September 2020 (NCT04530396).

## Replicating vectored vaccines in development for SARS‐CoV‐2

An alternative to replication deficient vectors is to use a live attenuated vector. Merck has recently acquired Themis, who have developed an attenuated measles vector vaccine approach using an attenuated strain of measles derived from the original 1954 vaccine strain. Themis have previously used this approach to develop a Chikungunya vaccine, which was safe and immunogenic [172]. In collaboration with Institut Pasteur and the University of Pittsburgh they are now running clinical trials with these vaccines (NCT04497298 and NCT04498247).

### Mucosal delivery of vectored vaccines

Live and vectored vaccines may lend themselves to mucosal delivery which may achieve better local immunity and has been used for other vaccines; for example, intranasal live attenuated influenza vaccine (LAIV). However, the enthusiasm for mucosal vaccines based on preclinical data has not always translated into clinical success. Symvivo is using oral delivery of a probiotic bacteria, *Bifidobacterium longum*, to deliver the spike transgene (NCT04334980). The Migal Galilee Research Institute have adopted an existing vaccine against infectious bronchitis virus (IBV), which has been used in a preclinical veterinary trial inducing humoral, cellular and mucosal immunity [173] to be delivered orally, but this is not yet in clinical trials. Beijing Wantai Biological Pharmacy and Xiamen University have recently registered a phase I clinical trial using an influenza viral vector (ChiCTR2000037782).

## Nucleic acid vaccines

Nucleic acid vaccines have been highlighted for their potential in pandemic situations due to their low cost and potential rapid development, although this potential has yet to be translated into a real‐world vaccine [174]. They utilize either plasmid DNA or RNA, encoding a target antigen. Following delivery of the vaccine, the nucleic acid is taken up by the cells and the encoded antigen is expressed. Conceptually, one facility can produce any required nucleic acid vaccine and production can be theoretically scaled‐up to meet pandemic level demands. The COVID‐19 pandemic will serve as an important test case for nucleic acid vaccines, with six RNA platforms and four DNA platforms currently in clinical trial.

## DNA vaccines

Most DNA vaccines are constructed from plasmids that contain prokaryotic sequences that support the plasmids’ propagation in *E. coli*, and a mammalian expression cassette that controls the expression of the target transgene in the vaccinated organism. The expression cassette contains an upstream promoter to drive transgene expression, a Kozak sequence, the inserted transgene and a 3′ polyadenylation (polyA) tail. Following delivery, the DNA vaccine is taken up by host cells local at the immunization site or by migrating APCs [175]. To induce an adaptive immune response the DNA must enter the cell nucleus. In transiting to the nucleus the DNA passes through the cytosol which is inflammatory, being sensed by intracellular pattern recognition receptors, for example STING1 [175] or TBK1 [176], inducing an innate immune response. The triggering of innate immunity is essential for promoting adaptive immunity to DNA vaccines. If APCs are transfected directly with a DNA vaccine, they will load vaccine‐encoded peptides onto both MHCI and MHCII molecules and activate T cells [177]. Transfected stromal cells will generate antigen, which will be encountered by APCs and B cells following antigen release from cell exosomes or apoptotic bodies. Transit of injected naked DNA to the nucleus is highly inefficient, with a large majority of the DNA failing to cross the cell membrane or nuclear envelope [178, 179]. To mitigate this loss, DNA vaccine programmes employ delivery platforms such as electroporation and bio‐injection.

### DNA vaccines – coronaviruses

Preclinical animal studies have demonstrated that DNA vaccines encoding the M, N, 3a or S proteins of the SARS‐CoV‐1 virus could elicit immune responses [180, 181, 182]. A multivalent DNA vaccine encoding S and M protein epitopes could protect from SARS‐CoV‐1 cytopathic effects. The S protein is the target of the only SARS‐CoV‐1 DNA vaccine to progress to Phase I clinical trial, delivered by bio‐injector, and it was safe and induced neutralizing antibody responses [183]. The leading DNA vaccine against MERS‐CoV (INO‐4700) was developed by Inovio. Phase I clinical trials were completed in 2019, with the vaccine showing a good safety profile and inducing humoral immunity and polyfunctional CD8^+^ T cell responses [184].

### DNA vaccines in development for SARS‐CoV‐2

The Inovio MERS INO4700 (GLS‐5300) vaccine that was due to be taken to Phase II clinical trials (NCT03721718) has now been redeployed as INO‐4800 (NCT04336410) to begin clinical trials for protection against SARS‐CoV‐2. In preclinical studies of the INO‐4800 vaccine, neutralizing antibody and T cell responses were observed in mice and blocking antibody responses in vaccinated guinea pigs [185] and macaques [186]. The Phase I trial (NCT04336410) is ongoing, but the data have not yet been published. Genexine, in South Korea (NCT04445389), Zydus Cadila in India (CTRI/2020/07/026352) and Osaka University in Japan (NCT04463472) have initiated Phase I trials of DNA vaccines.

## RNA vaccines

RNA vaccines are based on the same premise as DNA vaccines of expressing a vaccine antigen transgene in the host cell, but they are one step further along the expression pathway, skipping the transcription step. Unlike DNA vaccines, expression of RNA vaccines begins once they enter the cell cytosol, which can increase the efficiency of expression.

As with DNA vaccines, the presence of ‘foreign’ RNA is sensed in both the endosome and cytosol [187], giving RNA vaccines a self‐adjuvanting effect [188]. However, the early triggering of type I IFN responses can down‐regulate protein expression [189]. Modified nucleosides can be incorporated into the mRNA product to create a ‘silenced’ RNA vaccine that avoids detection by TLRs and does not trigger a type I IFN response [190, 191], but there is a balance between antigen expression from the vaccine construct and triggering enough inflammation to activate the immune response. This balance may be altered by the formulation to deliver the vaccine and can be different between different animal species, making predictions from preclinical studies difficult.

There are two primary types of RNA vaccine mRNA and self‐amplifying mRNA (saRNA). Non‐replicating mRNA vaccines are constructs engineered to encode the gene of interest, and typically have a 5′ cap, UTRs flanking the gene of interest and poly A tail. The 5′ cap is essential for mRNA to associate with the eukaryotic translation complex. UTRs are selected to optimize RNA protein expression, avoiding the inclusion of sequences that would hamper translation [192, 193]. mRNA vaccine constructs are made using bacteriophage‐derived RNA polymerases and NTPs to transcribe linearized DNA *in vitro*.

Self‐amplifying RNA vaccines are alphavirus‐derived RNA replicons modified to encode the antigen of interest in place of RNA structural proteins. The viral replicon also contains an open reading frame (ORF) that encodes four alphavirus non‐structural proteins (nsP1‐4) and a subgenomic promoter. The non‐structural proteins form an RNA‐dependent RNA polymerase (RDRP). The RDRP complex transcribes more copies of the vaccine in the transfected cell. As a result, saRNA vaccines express protein at higher levels and persist for longer than non‐replicating RNA [194].

### RNA vaccines in development for SARS‐CoV‐2

As it is a newer technology, RNA vaccines were not developed against SARS‐CoV‐1. Six RNA vaccines are in clinical trials for SAR‐CoV‐2.

Moderna fast‐tracked their candidate vaccine mRNA‐1273 and were first to begin clinical trials on 17 March 2020 with the National Institute of Health’s National Institute of Allergy and Infectious Diseases (NIAID) (NCT04283461). This Phase I study involved 45 patient volunteers, divided into three group cohorts, as a dose escalation: low (25 µg), middle (100 µg) and high (250 µg) in a prime boost. A preclinical study using the same vaccines was protective in mice against viral challenge [195]. The vaccine was immunogenic, with increasing antibody titres with increasing dose administered; of note, three individuals (of 15, 21%) in the 250‐µg group reported severe adverse events, with severity increasing after the second vaccination [196]. The vaccine is now in Phase II (NCT04405076) and Phase III (NCT04470427) trials, focusing on the 100‐µg dose.

BioNTech is collaborating with Pfizer to develop four S protein vaccine candidates. They are using a nucleoside modified mRNA. Phases I/II clinical trials are running in Germany (NCT04380701) and the United States (NCT04368728), with a multi‐site Phase III study planned. The vaccine induced both cellular and humoral responses in mice [197] and induced neutralizing antibody in the clinical study [198]. They have performed a further two clinical studies, observing similar responses in a second study with their initial construct (BNT162b1) which encodes a RBD trimer [199]. In a comparator study they observed similar levels of immunogenicity to a stabilised membrane anchored spike protein (BNT162b2), but with lower levels of reactogenicity [200]. Both CureVac and The People’s Liberation Army have also developed mRNA vaccines that are in clinical trials, but no results have been published as yet.

Imperial College London and its spin‐out social enterprise, VacEquity Global Health, are developing an saRNA vaccine encoding the S protein. Intramuscular injection with LNP formulation induced high neutralizing antibody titres in mice [103]. Tested doses of the preclinical vaccine ranged from 0·01 µg to 10 µg, with a boost of the same dose at week 4 post‐vaccination. Human trials of the vaccine with 420 participants started in June 2020 (ISRCTN17072692). Arcturus, based in Singapore, are also developing an saRNA vaccine encoding a prefusion spike, which is in Phase I clinical trial (NCT04480957).

## Other aspects concerning vaccines

### Adjuvants

Protein vaccines can have low levels of immunogenicity. This can be boosted by adjuvants [201]. Adjuvants enhance the immune response through multiple mechanisms, causing a depot effect; up‐regulating the production of chemokines and cytokines; enhancing the cellular recruitment to site of injection; increasing antigen uptake and presentation by APCs and increasing inflammasome activation [202]. Adjuvants can also tailor the immune response, guiding it towards producing the most effective form of immunity against the specific pathogen being vaccinated against [203, 204, 205]. A range of adjuvants have been proposed for use with SARS‐CoV‐2 protein vaccines. These include Advax, alum, AS03 (GSK), Matrix‐M (Novavax), CpG (Dynavax) and MF59 (CSL).

### Formulation and delivery

Additional components are also included with nucleic acid vaccines to enhance uptake and immunogenicity. Nucleic acids are combined with a range of formulations, including lipid nanoparticles (LNPs), liposomes and polyplexes. Such formulations are essential for RNA vaccines, as ‘naked’ RNA is susceptible to being degraded by extracellular RNAses which will prevent efficient cell uptake. LNPs have previously been used for other RNA therapeutics and Moderna, Imperial College London, Arcturus, Curevac and BioNTech vaccines all utilize this technology. The stability of these formulations can be a concern and may necessitate vaccine storage at a lower temperature which might, in turn, impact access to the vaccine. For DNA vaccines, delivery devices are often used to increase uptake. Inovio uses an electroporation device (Cellectra^®^ 2000), which delivers an electric current to the site of injection: in a study on acceptability, acute pain (six of 10 on the VAS score) was recorded for the first 5 min after immunization, but this receded [206]. A similar effect was observed in a study using a different electroporation device [207]. Genexine are also using electroporation in their trial, but they are also comparing with a needle‐free biojector. Biojector devices have been shown to increase the antibody response to DNA vaccines [208, 209].

### Inducing non‐specific immunity with vaccines

One of the more experimental approaches proposed to reduce the impact of COVID‐19 has been the use of other live vaccines as non‐specific vaccines [210]. This has been proposed for bacille Calmette–Guérin (BCG), oral polio vaccine [211] and measles, mumps and rubella (MMR) [212]. The proposed mechanism is described as trained immunity, where exposure to one agent alters the epigenetic profile of innate immune cells, potentially increasing the production of cytokines. In preclinical models, BCG pretreatment has been shown to reduce influenza viral titres [213]. Early ecological data (in April 2020) suggested that countries with mandatory BCG vaccination had reduced mortality from COVID‐19, but this analysis has a number of issues, mainly associated with demographics and the timing of when the virus reached different countries [214]. A more recent study has supported this protective effect [215]. Remarkably, a number of randomized clinical trials have been set up to directly test whether BCG can reduce the burden of COVID‐19.

## Pros and cons of different platforms

The COVID‐19 pandemic has led to a surge of different vaccines being rapidly moved to clinical trials. A number of these vaccines have been around for several years as promising preclinical platforms, but not necessarily been attractive enough to generate funding to support human trials. Each of the approaches has advantages and disadvantages (Table 4): which aspects are the most important will only be identified following efficacy studies. Live attenuated vaccines have a long track record of safety and efficacy, but they may not be feasible in the current pandemic due to the length of time it takes to generate a candidate and test for attenuation. Inactivated vaccines also have a long track record of protective efficacy, and they have the advantage that they are fast to generate; however, they require high‐containment facilities to generate the virus stock. There is also a concern about vaccine‐induced immunopathology with an inactivated vaccine, which has been seen for some other respiratory viruses and in preclinical models of SARS‐CoV‐1; whether this is the case for inactivated SARS‐CoV‐2 vaccines will only be seen after larger and longer Phases II/III trials. Recombinant protein vaccines have been in use since the 1980s; they are a more targeted approach than using a whole virus, which may focus the immune response on a key antigen, but this may lose some breadth of protection. Protein candidates were somewhat slower to enter clinical trials but may have a faster route to licensure, being a more known product than newer vaccines. One challenge is to use the correct conformation of the protein, Spike is metastable and may be less protective if used in a post‐fusion form. One peptide vaccine has registered a clinical trial (NCT04527575) from the Vector institute in Koltsovo, Russia; it is adjuvanted with alum.

**Table 4:** Advantages and disadvantages of different vaccine platforms

Vaccine	Advantage	Disadvantage
Live Attenuated	Good track record	Risk of reversion to pathogenic form
	Manufacturing capacity	Slow to develop new versions
		Risk of infection in immunocompromised patients
		May require BSLIII to generate and test
Inactivated vaccines	Fast to generate	Need live virus and facility to grow large amounts
	Long track record	Risk of vaccine‐enhanced disease
Protein vaccines	Safe	Potentially poorly immunogenic without adjuvant
Including VLP	Very common platform	Risk of wrong conformation
		Slow and more expensive manufacture
Peptide	T cell response	Risk of T cell enhanced disease
		Poorly immunogenic
aAPC	T cell response	Requires cell manufacture, issues of scale up
		Impractical
Viral vectored vaccines	No need to grow live virus	Pre‐existing anti‐vector immunity
	Fast to generate	T cell focused response, lower antibody induction
	Safe track record	Requires low temperature (‐80°C) storage
		Replicating vectors not suitable for immunocompromised patients
DNA vaccines	Fast to generate	Poor track record of immunogenicity in human trials
	Safe	
	Thermostable	
mRNA vaccines	Fast to generate	New platform: Not yet used in human efficacy study
	Translation in cytosol	Unstable
		Needs formulation
saRNA vaccines	Fast to generate	New platform: Previously not been in human clinical trial
	Requires lower dose than mRNA	Unstable
	Potential for mass production	Needs formulation

The cellular‐based approaches, using aAPC, do not seem to be practical for wide‐scale rollout. Nucleic acid vaccines, both DNA and RNA, have much potential in terms of speed of response and scale‐up: this outbreak will be an important test for whether they can deliver on their promise. DNA vaccines have historically been less immunogenic than other platforms, although with alternate delivery devices that may be overcome. RNA vaccines have not been widely tested for infectious diseases; this is the first time an saRNA vaccine has been trialled. RNA may have a slight issue concerning heat‐stability, necessitating –80˚C storage. Viral vectored vaccines are the furthest ahead in clinical trials, with three candidates in later Phase clinical trials. They are known to be safe, but may be reactogenic at higher doses. Historically, these approaches have had mixed results for efficacy and one concern is pre‐existing immunity against the vector, especially when a human viral‐derived vector such as Ad5 is used. It will be of great interest to see which platforms and candidates are protective in efficacy trials.

As of September 2020, the furthest advanced candidates have completed Phase I trials (Table 3), although so far not all the organizations involved have published data from completed trials. All the data published so far indicate that the vaccines are safe, but there are more adverse events at higher doses: both the Moderna [196] and BioNTech [198] vaccines had severe adverse effects at the highest doses, leading to a lower dose in later studies; some severe adverse effects were also recorded in the Cansino [166, 167] and University of Oxford [165] vectored vaccine trials. The vaccines all appear to be immunogenic, although it is hard to compare directly, as different groups will have used subtly different ELISA and neutralization assays. A further complication for comparison is when data have been published as press releases rather than peer‐reviewed papers. Ultimately, vaccine efficacy in a randomized trial is the most important issue, but here again, different primary end‐points are being explored. Some studies are looking at reduction of disease, while others are looking at reduction of confirmed infections.

## Translation into a real‐world vaccine

The COVID‐19 crisis represents an opportunity for several experimental vaccine platforms to progress to clinical trials. However, there are considerations between a promising preclinical candidate and a global vaccination campaign, including trials, regulation and manufacture.

### Clinical trials during a pandemic

Clinical vaccine trials conventionally undergo four broad Phases, from early safety in small numbers of volunteers (Phase I) to wide‐scale post‐licensure monitoring (Phase IV). Usually, each of the Phases take months or even years to complete before moving on. In a pandemic setting, there is need to speed up the transition between Phases; this has been achieved with co‐operation of regulatory agencies and research ethics committees. Additionally, Phases can be merged, with planning of Phases II/III trials initiated before the Phase I trial has even begun. There is the potential that data obtained from Phase I will mean that planned later‐Phase trials are cancelled due to safety concerns or futility. Ultimately, pushing a vaccine through the different stages of a clinical trial does not negate the need for complete safety data sets to be collected, but close co‐operation with the researchers and regulators can accelerate a progress that could reduce 10 years to 18 months. One consideration is that due to the accelerated time‐scale, post‐licensure monitoring will be extremely important. Another issue concerns the sample size required for efficacy studies; as cases fall, larger studies will be necessary or an alternate trial design. A ring trial was used in Ebola [216], which allowed efficacy to be assessed in a few individuals.

### Manufacturing

One of the major hurdles to a vaccine relieving the COVID‐19 pandemic is manufacturing enough doses to achieve global herd immunity. The number of doses needed to achieve global coverage depends upon the regime used, but is potentially as many as 16 billion (assuming a prime boost regime with some contingency). To ensure licensure and prequalification status, good manufacturing process (GMP) standards must be upheld during up‐scaled manufacturing and clinical studies. Manufacturing a vaccine at a global scale in the time‐frame required is a unique challenge. Vaccines that are not only safe and effective but also highly scalable, to produce millions or even billions of doses, would be the most desirable tool for curbing the pandemic. Logistics are a key consideration, including access to components to manufacture the vaccines – for example, nucleic acid vaccines require nucleotide triphosphates (NTPs), which are also in high demand for the diagnostic tests. The vaccines also require plant and materials to fill and finish the final product; one bottleneck is exactly that: glass bottles. In parallel with the accelerated clinical trials, accelerated manufacturing scale‐up is required. This telescoped manufacturing process means that investment in the next step is being made before the results of the previous step are known. This has considerable financial risk, especially in terms of setting up the necessary manufacturing plant if it cannot be repurposed, either for other pandemic vaccines or other biologicals.

Funding is a critical part of the vaccine development process. It remains to be seen whether the total costs of research, development and licensure of any novel vaccine platforms for SARS‐CoV‐2 is comparable to traditional vaccines, such as Dengvaxia, which costed approximately $1.5 billion until licensure [217]. A range of funding mechanisms have supported vaccine development. One of the major bodies co‐ordinating the funding is CEPI, which has received funding from multi‐national sources, including governments and charities. Other vaccine candidate teams are being supported by their governments ‘fast‐tracking’ their candidates through clinical trials and streamlining their manufacturing. For example, Operation Warp Speed in the United States, is supporting six candidates from AstraZeneca (AZD1222), Moderna (RNA), Pfizer (RNA with BioNTech), Merck (vectored vaccine with Themis), Johnson and Johnson (vectored vaccine) and Novavax (recombinant protein). A UK Vaccine Task Force was announced on 20 April 2020 [218] to support UK‐based candidates and reviewing government regulations to facilitate rapid and safe vaccine trials.

Several vaccine candidates have never progressed past Phase I before, therefore manufacturing GMP material at scale poses new challenges. Many of the vaccines are being developed by either academic groups or small‐ to medium‐sized biotech companies, neither of which necessarily have capacity to manufacture at a large scale. One approach is outsourcing to contract manufacturing organizations (CMOs), which can have licensing complications. Some companies have invested in manufacturing capacity; for example in July 2018, Moderna opened a manufacturing facility in Norwood (USA) [219], which produce RNA up to the gram scale. However, they still need to work with external companies to complete formulation protocols. New manufacturing facilities are also being constructed elsewhere, including the Vaccines Manufacture and Innovation Centre (VMIC) in the United Kingdom, which has been accelerated and is planned to open in mid‐2021, and Valneva opened an expanded BSLIII facility in Scotland in August 2020.

Larger companies may have more experience and capacity of manufacturing; for example, Janssen and AstraZeneca are making adenovirus vectors and Sanofi is making a protein vaccine. In the initial stages of the outbreak there was relatively little publicized activity from the larger pharmaceutical companies, with most of the attention on smaller biotechs and academic groups. It is not clear why this was the case. One possible reason could have been exemption from liability, although vaccine manufacturers are exempt in the United States under the 2005 Public Readiness and Emergency Preparedness, or PREP Act.

Integrating national funding programmes with equitable global access to vaccines is vital. There is a concern that wealthy countries will monopolize initial production runs of vaccines, with preorders outstripping manufacturing capacity. Alternative models of licensure, social enterprise and spoke and hub manufacture may be necessary. For example, the AstraZeneca/Oxford vaccine has been licensed to the Serum Institute of India, the largest global manufacturer of vaccines by doses produced, and Imperial College London have established a social enterprise company to enable equitable access.

### Regulation of SARS‐CoV‐2 vaccines

Vaccines require regulation to be introduced as a licensed product. There are a range of national and international bodies which cover this process; for example, any product trialled in the United Kingdom will be considered for approval by the Medicines and Healthcare products Regulatory Agency (MHRA). The product is then considered for prequalification by the WHO for multi‐national distribution, which has supported the regulation and distribution of vaccines for 33 years [220]. This process can normally take a substantial amount of time; however, during the COVID‐19 pandemic manufacturers and regulators are striving towards the delivery of a vaccine that is safe and effective within 18 months from February 2020. There is a precedent for accelerated licensure in the context of a pandemic, as seen with the approval of rVSV‐ZEBOV [221] as a vaccine for Ebola. The WHO supported the accelerated regulatory approval for rVSV‐ZEBOV‐GP using an expedited prequalification review following its receiving conditional marketing authorization from the European Medicines Agency (EMA) [222, 223]. The WHO has issued guidance and recommendations for the regulation of SARS‐CoV‐2 vaccines [220].

## Lessons learned

The issues faced during this pandemic and the vaccine platforms being developed to address it will be invaluable for future outbreak control. While, at this stage, it is not possible to say which platform is best, and what works best for one infection may not be best for all infections or for all populations. One of the biggest considerations of all the platforms is speed into trials *versus* ability to deploy the vaccine. While some of the high‐speed platforms, for example RNA, have entered into Phase I trials faster than other approaches, their lack of track record means that approaches for global scale‐up are potentially slower. Older approaches may leapfrog the newer platforms in the scale‐up and manufacturing stage. One observation of interest is the speed with which one of the oldest technologies for viral vaccines, inactivation, has been able to move forwards. Prior to COVID‐19, much of the attention for pandemic vaccine preparedness was on newer technologies; however, of the candidates in Phase III in August 2020, two of four are inactivated vaccines. Safety and efficacy data from these large studies will be critical. Another consideration is that while distributed global manufacture is effective and appropriate for routine scheduled vaccination, local surge manufacturing capacity for new vaccines is important. This capacity may have to be maintained at a loss for large amounts of time, unless alternative commercial contracts can be found for the same facility that can then be replaced at short notice. This reflects a broader consideration that investment in public health, which may appear expensive to begin with, can save a considerable amount in the long term; one study estimated that every £1 spent on public health saves £14 in return [224]. Another consideration is for greater standardization of assays and end‐points. Comparisons of the different trials has been made significantly harder by the use of different methodologies. This is part of the broader global context of a true pandemic. Collaborative worldwide action is required to control the virus, which necessitates leadership and a willingness to share by countries with more developed scientific research programmes. Ultimately, while lessons will be learned from this pandemic, the next one will be different and sticking rigidly to a plan that controlled this coronavirus will not necessarily work for a different virus, as the German strategist Helmuth von Moltke ‘sort of’ said: ‘No plan survives contact with the enemy’.

## Conclusion

It is still far too early to know what the best approach will be to control COVID‐19 with vaccines. Speculating as to which vaccine platform is ‘best’, while academically enjoyable, is not of value here. That so many platforms, both new and old, are moving into efficacy study makes this an extremely exciting time for vaccinology. The outbreak will certainly be a test case for the novel vaccine platforms, particularly nucleic acid vaccines, which have promised much to date but not been licensed for human use. One issue is whether vaccines will play a role in reducing the burden of the pandemic. Even at maximum speed, the first efficacy trials will start 9 months after the start of the pandemic and the first licensed doses are unlikely to be ready for 18 months, by which time the virus will have caused a large wave of mortality and a larger wave of global disruption. Important questions remain (Box 1) regarding what is a successful vaccine, how should it be deployed and who should be prioritized. These will depend in part upon the results of the efficacy studies, although the WHO has produced some draft guidance [225]. Overall, it has been a remarkable chapter in vaccine development, with widespread collaboration and partnership in a race against the virus.

Box 1 **Questions remaining**

**Table:** 

What is the best type of protection?
Is sterilizing immunity the only approach or would disease reduction, without altering disease transmission work?
How to achieve equitable global distribution?
Is human challenge part of the pathway to a licensed product?
What is the risk of immunopathology?
How long will protection last after natural infection or vaccination?
Can better immunogenicity be achieved with vaccination than after natural infection?

## Disclosures

H. M. C. and K. M. P. are investigators on the Imperial College London vaccine program. B. F. P. works in part for VacEquity Global Health. K.M.P. is an invited member of the Sanofi influenza vaccine advisory panel 2020.
